# Association of circulating levels of nicotinamide phosphoribosyltransferase (NAMPT/Visfatin) and of a frequent polymorphism in the promoter of the *NAMPT* gene with coronary artery disease in diabetic and non-diabetic subjects

**DOI:** 10.1186/1475-2840-12-119

**Published:** 2013-08-22

**Authors:** Pedro Saddi-Rosa, Carolina Soares Oliveira, Felipe Crispim, Fernando MA Giuffrida, Valter Correia de Lima, José Gilberto Vieira, Alessandro Doria, Gilberto Velho, André Fernandes Reis

**Affiliations:** 1 Federal University of São Paulo (UNIFESP), Diabetes Center, Rua Pedro de Toledo 910, São Paulo CEP 04039-002, SP Brazil; 2 CEDEBA, Av. Antonio Carlos Magalhães, S/N, Salvador CEP 41820-000, BA, Brazil; 3 Cardiology Unit, UNIFESP, Rua Napoleão de Barros, 715 CEP, Térreo. São Paulo 04039-002, SP Brazil; 4 Research Division, Joslin Diabetes Center, One Joslin Place Boston 02215, MA, USA; 5 INSERM, Research Unit 695, 16 rue Henri Huchard, Paris 75018, France

**Keywords:** NAMPT, Visfatin, Coronary artery disease, Type 2 diabetes, rs9770242

## Abstract

**Background:**

Nicotinamide phosphoribosyltransferase (NAMPT) is the limiting enzyme in one of pathways of synthesis of Nicotinamide Adenine Dinucleotide, a redox coenzyme. NAMPT is considered as an insulin-mimetic factor and a potential regulatory factor in inflammatory and immune processes. Associations of circulating NAMPT levels with cardiovascular disease (CVD) and insulin resistance have been reported. We investigated association of circulating NAMPT levels and the rs9770242 *NAMPT* gene polymorphism with coronary artery disease (CAD).

**Methods:**

We studied 594 Brazilian subjects undergoing a coronary angiography (49% of whom had type 2 diabetes). CAD, defined as stenosis greater than 50% in one major coronary vessel or branch, was observed in 68% of subjects. Genetic studies were also performed in 858 North-American Non-Hispanic White subjects with type 2 diabetes (49% with CAD).

**Results:**

We observed an interaction between glycemic and CAD status on the comparison of NAMPT levels by CAD status. NAMPT levels were higher in type 2 diabetic patients with CAD as compared to those without CAD: 5.27 ± 2.93 ng/ml vs. 4.43 ± 2.94 ng/ml, p = 0.006 (mean ± SD). NAMPT levels were not significantly different in non-diabetic subjects with or without CAD. The T-allele of rs9770242 was associated with CAD in the Brazilian cohort (OR 1.46, 95% CI 1.06 - 2.01, p = 0.02) while no association was observed in the North-American cohort.

**Conclusions:**

Our data suggest that circulating NAMPT levels are associated with CAD in type 2 diabetic patients. *NAMPT* rs9770242 polymorphism may be associated with CAD in some populations.

## Introduction

The adipose tissue secretes several adipokines, including adiponectin, leptin, resistin and, of more recent discovery, nicotinamide phosphoribosyltransferase (NAMPT) [1]. It was suggested that these molecules could explain, at least in part, the link between obesity, insulin resistance, beta-cell dysfunction, endothelial dysfunction, atherosclerosis and cardiovascular disease (CVD) [1-6].

NAMPT, formerly known as visfatin or pre-B cell colony enhancing factor (PBEF), is expressed throughout the body. It is secreted mainly by adipocytes and macrophages [5], but also by human pancreatic beta cells where its secretion is upregulated by glucose [7]. NAMPT is the limiting enzyme in the mammalian salvage pathway of synthesis of Nicotinamide Adenine Dinucleotide (NAD^+^), an ubiquitary coenzyme involved in redox reactions. NAMPT is considered as an insulin-mimetic factor and a potential regulatory factor of inflammatory and immune processes [1,5,8]. Associations of circulating NAMPT levels with several inflammatory conditions and metabolic alterations have been reported. For instance, some studies have shown associations of circulating levels of NAMPT with CVD, as well as with surrogates of insulin resistance [3,9-11]. The human *NAMPT* gene maps to a region on chromosome 7q22.2 previously found to be linked to insulin resistance syndrome-related phenotypes in nondiabetic Mexican-Americans [12]. In addition, several frequent polymorphisms (minor allele frequency >0.05) in the *NAMPT* gene were reported to be associated with a variety of physiological and pathological phenotypes including obesity, dyslipidemia, low-grade inflammation, and type 2 diabetes [13-15]. The T-allele of the promoter variant rs9770242 was shown to be associated with higher fasting plasma glucose and insulin levels [14] and with higher visceral/subcutaneous ratio of *NAMPT* mRNA expression in adipose tissue [15]. In different populations, this SNP is in perfect or moderate linkage disequilibrium (LD) with other SNPs in the 5′ region located in the putative promoter region (−948 G > T, rs1319501) [14,15].

In the present study we investigated associations of circulating levels of NAMPT with coronary artery disease (CAD) and related phenotypes in Brazilian subjects with high cardiovascular risk, with or without type 2 diabetes. We also studied the association between the rs9770242 SNP and CAD in this Brazilian cohort and in an independent cohort of North-American non-Hispanic white subjects with type 2 diabetes.

## Methods

### Subjects: Brazilian cohort

A total of 594 consecutive patients who underwent coronary angiography at Hospital São Paulo-Brazil (UNIFESP University Hospital) were enrolled in the present study, as previously described [16]. In brief, subjects were 60 ± 10 years old, 56.7% of them were men and 49% had type 2 diabetes. Patients were referred to cardiac catheterization by their physicians for reasons such as stable angina, positive stress test, non-ST-elevation acute coronary syndrome. For the purpose of this study, CAD was defined as any stenosis greater than 50% in at least one major coronary vessel or branch observed during coronary angiography. Diabetes mellitus was defined according to the American Diabetes Association criteria [17]. Arterial hypertension was defined as systolic blood pressure (SBP) ≥140 mmHg and/or diastolic blood pressure (DBP) ≥90 mmHg, or current use of antihypertensive medication and history of hypertension. Subjects were considered to have dyslipidemia either if levels of LDL-cholesterol were ≥4.14 mmol/l, HDL-cholesterol ≤1.04 mmol/l, triglycerides ≥2.26 mmol/l, or in the presence of use of lipid lowering medications (statin/fibrates). Study subjects, individually and/or as a group, had mixed ethnic background (African, Amerindian, Asian, and European Caucasian of several different countries of origin), reflecting the Brazilian population [18]. All participants gave written informed consent. The study was approved by the ethics committee of UNIFESP.

### Subjects: North-American cohort

We studied 858 Non-Hispanic white people. Clinical characteristics of a subset of this population have been previously described [19]. In brief, CAD positive case patients (n = 424) were a random sample of patients with type 2 diabetes who had a stenosis greater than 50% in a major coronary artery or a main branch, documented by cardiac catheterization at the Beth Israel Deaconess Medical Center (BIDMC) between 2001 and 2008. CAD-negative controls (n = 434) were randomly selected from among 903 Joslin patients as fulfilling the following criteria: current age between 55 and 74 years; type 2 diabetes for five years or more; negative cardiovascular history and normal response to an exercise treadmill test. The study protocol and informed consent procedures were approved by the Joslin Committee on Human Studies and the BIDMC Committee on Clinical Investigations.

### Clinical and biological procedures

In the Brazilian set, a blood sample was drawn after an overnight fast for analysis of plasma glucose, plasma insulin, HbA1c (HPLC, reference value: 4.5-6.0%), lipid profile (total serum cholesterol, LDL-cholesterol, HDL-cholesterol, and serum triglycerides), TSH and creatinine. Glomerular filtration rate was estimated using the Modification of Diet in Renal Disease study equation [20]. Plasma NAMPT levels were measured using commercial ELISA kits (EK-003-80, Phoenix Pharmaceuticals). Intra- and interassay coefficients of variation were respectively <10% and 14.8% (sensitivity: 2.17 ng/ml). Total adiponectin levels were measured in plasma samples using commercial ELISA kits (EZHADP-61 K, Millipore, Saint Charles, MO). Intra- and interassay coefficients of variation were respectively 7.4% and 10.6% (sensitivity: 0.78 ng/ml).

### DNA studies

In both cohorts, DNA was extracted from peripheral blood samples by standard procedures. The choice of *NAMPT* promoter rs9770242 was based on results from previous studies suggesting that it might modulate NAMPT mRNA expression in visceral and subcutaneous fat depots [15]. The SNP was evaluated using a Taqman SNP Genotyping Assay by Applied Biosystems. Genotyping was repeated in 20% of subjects from the Brazilian cohort using an ABI3131XL genetic analyzer; concordance rate was >99% and total genotype success rate was 97%. In the North-American study, genotyping quality was assessed by including six blinded duplicate samples in each 96-well assay. The average agreement rate was greater than 99%.

### Statistical analyses

Differences between groups were assessed by Pearson’s chi squared test, ANOVA and ANCOVA. Interaction between glycemic status (type 2 diabetes: yes/no) and CAD status in the comparison of NAMPT levels were assessed by including in the ANCOVA model a “crossed” compound covariate (glycemic status/CAD status). Stratification by glycemic status was then performed by nesting the CAD status variable within the glycemic status variable in the ANCOVA. This results in the computation of statistical effects for subjects with or without diabetes separately, and adjusted for multiple comparisons due to the stratification by glycemic status.

Associations with CAD were assessed by logistic regression analyses and odds ratios with their 95% confidence interval were computed. Adjustments for clinical and biological parameters were carried out by including these parameters as covariates in the regressive model. For all analyses, data were log-transformed when the normality of the distribution was rejected by the Shapiro-Wilk W test. The power to detect associations of rs9770242 with CAD in both cohorts was >90%, for odds ratio ≥1.5 and alpha = 0.05. Statistical analysis was performed with the JMP software (SAS Institute Inc., Carey, NC).

## Results

### Plasma NAMPT levels and CAD

The coronary angiogram was positive for CAD in 68% of subjects in the Brazilian cohort. Clinical characteristics of subjects with or without CAD are shown in Table 1. We observed an interaction between glycemic and CAD status on the comparison of plasma NAMPT levels by CAD status (p = 0.003 for the interaction). Plasma NAMPT levels were higher in type 2 diabetic patients with CAD as compared to those without CAD: 5.27 ± 2.93 ng/ml vs. 4.43 ± 2.94 ng/ml, p = 0.006 (mean ± SD, ANCOVA with log-transformed data, adjusted for age and sex). The difference between groups remained significant when the comparison was further adjusted for BMI, dyslipidemia, arterial hypertension and tobacco smoking (data not shown).

**Table 1:** Brazilian cohort – clinical characteristics of participants by CAD status

	**With CAD**	**Without CAD**	**p**
Subjects (n)	406	188	
Sex: Male (%)	63	50	0.0005
Age (y)	60 ± 11	57 ± 11	0.0001
Diabetes mellitus (%)	48	52	0.35
Fasting plasma glucose (mmol/l)	7 ± 2.78	6.39 ± 1.89	0.03
HbA1c (%)	6.8 ± 1.6	6.6 ± 1.5	0.11
BMI (kg/m^2^)	27.1 ± 4.6	28.0 ± 5.0	0.06
Dyslipidemia (%)	95	86	0.01
Triglycerides (mmol/l)*	1.47[1.00-1.95]	1.36[0.89-1.84]	0.0002
LDL Cholesterol (mmol/l)	2.59 ± 0.93	2.67 ± 0.88	0.40
HDL Cholesterol (mmol/l)	0.98 ± 0.28	1.09 ± 0.28	0.0001
eGFR(ml/min)	84 ± 24	89 ± 28	0.05
Hypertension (%)	91	90	0.51
Systolic BP (mmHg)	140 ± 24	136 ± 20	0.09
Diastolic BP (mmHg)	80 ± 14	79 ± 13	0.54
Tobacco smoking (%)	57	42	0.003

Data expressed in mean ± SD, except *expressed in median [interquartile range]. *CAD* coronary artery disease, *HbA1c* glycated hemoglobin, *eGFR* estimated glomerular filtration rate. Differences between groups were assessed by Pearson’s chi squared test and ANOVA.

The prevalence of CAD in type 2 diabetic patients by tertiles of plasma NAMPT was 57% (T1), 67% (T2) and 75% (T3). Logistic regression analyses showed a positive association of the highest tertile of plasma NAMPT with CAD: OR 2.09, 95% CI 1.11 – 4.01, p = 0.02 (T3 vs T1); OR 1.62, 95% CI 0.83 – 3.19, p = 0.15 (T3 vs T2) and OR 1.29, 95% CI 0.68 – 2.44, p = 0.42 (T2 vs T1; all comparisons adjusted for sex and age). NAMPT levels were not significantly different in non-diabetic subjects with or without CAD: 4.81 ± 3.00 ng/ml vs 5.25 ± 2.97 ng/ml, respectively (p = 0.14).

Clinical characteristics of subjects by tertiles of plasma NAMPT and glycemic status are shown in Table 2. Non-diabetic subjects in the highest tertile had lower BMI, lower levels of HbA1c, LDL cholesterol and triglycerides, and higher levels of total adiponectin. These clinical parameters were not significantly different across tertiles of NAMPT in subjects with type 2 diabetes, except triglyceride levels that were also lower in subjects in highest tertile. NAMPT levels were similar in subjects with or without type 2 diabetes (4.97 ± 3.07 vs 4.99 ± 2.77, respectively, p = 0.92; mean ± SD, ANCOVA with log-transformed data, adjusted for sex, age and BMI) and no association of NAMPT tertiles with diabetes was observed (data not shown).

**Table 2:** Clinical characteristics by tertiles of plasma NAMPT and glycemic status in Brazilian cohort

	**Subjects without type 2 diabetes**			**p**	**Type 2 diabetes subjects**			**p**
	**T1**	**T2**	**T3**		**T1**	**T2**	**T3**	
N	98	106	97		100	92	101	
Plasma NAMPT(ng/ml)	3.06 ± 0.74	5.02 ± 0.56^a^	8.87 ± 2.92^ab^	<0.0001	3.10 ± 0.73	5.00 ± 0.50^a^	8.67 ± 2.90^ab^	<0.0001
	(0.57 – 4.13)	(4.14 – 6.05)	(6.08 – 19.92)		(0.93 – 4.13)	(4.14 – 6.03)	(6.08 – 20.18)	
Age (y)	57 ± 10	59 ± 11	59 ± 11	0.31	59 ± 9	61 ± 10	64 ± 10^a^	0.01
Sex (male)	58	58	67	0.32	46	61	51	0.11
BMI (kg/m^2^)	26.8 ± 4.3	25.6 ± 4.2	25.3 ± 4.4^a^	0.04^c^	28.5 ± 5.3	27.5 ± 4.6	28.2 ± 4.5	0.31^c^
Waist circumference (cm)	97 ± 12	90 ± 14	92 ± 12	0.05^c^	102 ± 12	98 ± 12	99 ± 12	0.19^c^
Fasting Plasma Glucose (mmol/l)	5.6 ± 0.6	5.5 ± 0.6	5.4 ± 0.5	0.07	8.6 ± 3.7	8.1 ± 3.0	7.9 ± 2.6	0.38
HbA1c (%)	5.8 ± 0.4	5.9 ± 0.4	5.7 ± 0.4^ab^	0.004	7.7 ± 1.7	7.6 ± 1.6	7.8 ± 1.8	0.78
LDL cholesterol (mmol/l)	2.77 ± 0.89	2.86 ± 0.93	2.59 ± 0.95	0.05	2.40 ± 0.79	2.58 ± 1.01	2.55 ± 0.92	0.62
HDL cholesterol (mmol/l)	0.98 ± 0.25	1.04 ± 0.28	1.01 ± 0.28	0.45	1.04 ± 0.28	0.98 ± 0.29	1.00 ± 0.30	0.29
Triglycerides (mmol/l)	1.85 ± 1.53	1.54 ± 0.69	1.38 ± 0.68^a^	0.002	1.96 ± 1.22	1.94 ± 1.09	1.67 ± 0.94^a^	0.05
Total adiponectin (μg/ml)	8.8 ± 6.1	10.4 ± 6.9	11.2 ± 7.8^a^	0.006^c^	8.4 ± 5.5	8.3 ± 8.1	8.5 ± 6.2	0.82^c^
Arterial hypertension (%)	87	81	87	0.44	96	100	95	0.15
eGFR (ml/min)	87 ± 21	87 ± 25	86 ± 22	0.89	84 ± 24	82 ± 29	84 ± 28	0.56
Prevalence of CAD (%)	77	64	70	0.15	57	67	75	0.02
Antihypertensive medication (%)								
ACEi	50	44	42	0.52	61	71	65	0.31
ARB	12	14	13	0.92	17	10	10	0.23
BB	41	48	40	0.47	57	52	54	0.48
Antidiabetic medication (%)								
MTF	--	--	--	--	46	45	41	0.79
SU	--	--	--	--	27	22	23	0.75
INS	--	--	--	--	22	20	33	0.10

Data expressed as mean ± SD (and range, for plasma NAMPT levels). Statistics for quantitative parameters are ANOVA or ANCOVA with log-transformed data. Tukey Kramer HSD test following ANOVA/ANCOVA: significantly different (p < 0.05) from T1 (a) or T2 (b). Comparison adjusted for age and sex (c). *eGFR* estimated glomerular filtration rate, *UAE* urinary albumin excretion rate, *ACEi* angiotensin converting enzyme inhibitor, *ARB* angiotensin receptor blocker, *BB* beta-blocker *MTF* metformin, *SU* sulfonylurea, *INS* insulin.

### NAMPT rs9770242 and CAD

Genotype frequencies of rs9770242 by CAD status in both cohorts are shown in Table 3. The prevalence of CAD by genotype in the Brazilian cohort was 55% (GG), 66% (TG) and 70% (TT) suggesting a possible codominant effect for the T-allele. We observed in the Brazilian cohort a nominal association of the T-allele with CAD (OR 1.36, 95% CI 1.01 – 1.86, p = 0.05, in a codominant model, adjusted for sex, age and glycemic status). A more robust association was observed with further adjustment for BMI, blood pressure, dyslipidemia, and NAMPT levels (OR 1.46, 95% CI 1.06 – 2.01, p = 0.02). There was trend towards higher plasma NAMPT levels in carriers of the risk T-allele: 4.82 ± 1.99 (GG), 5.88 ± 2.87 (TG), 5.49 ± 2.81 ng/ml (TT), mean ± SD, p = 0.11 adjusted for age and sex. No genotype association with CAD was observed in the North-American cohort (OR 1.02, 95% CI 0.76 – 1.22, p = 0.85, in a codominant model for the T-allele, adjusted for sex and age). Similarly, no association was observed when considering only those cases (n = 190) that had a previous history of myocardial infarction (OR 1.09, 95% CI 0.78-1.32, p = 0.51).

**Table 3:** Genotype and allele frequencies of rs9770242 by CAD status

	**N**	**rs9770242**				**OR (95% CI)**	**P**
		**MAF**	**TT**	**TG**	**GG**		
Brazilian cohort							
Without CAD	188	0.245	0.582 (n = 109)	0.351 (n = 66)	0.067 (n = 13)	1	
With CAD	406	0.201	0.639 (n = 259)	0.323 (n = 131)	0.038 (n = 16)	1.36 (1.01 – 1.86)^a^	0.05
						1.46 (1.06 – 2.01)^b^	0.02
North-American cohort							
Without CAD	434	0.247	0.562 (n = 244)	0.383 (n = 166)	0.055 (n = 24)	1	
With CAD	424	0.245	0.543 (n = 230)	0.424 (n = 180)	0.033 (n = 14)	1.02 (0.76 – 1.22)^c^	0.73

OR (odds ratio) in a codominant model for the T-allele determined in logistic regression analyses.^(a)^Adjusted for sex, age and glycemic status; ^(b)^adjusted for sex, age, glycemic status, BMI, blood pressure, dyslipidemia and NAMPT levels; ^(c)^adjusted for age and sex. MAF: minor allele frequency. Hardy-Weinberg equilibrium: Brazilian cohort, without CAD (p = 0.49), with CAD (p = 0.91); North-American cohort, without CAD (p = 0.54), with CAD (p = 0.003).

## Discussion

NAMPT has emerged in the last few years as a novel adipokine potentially implicated in the pathogenesis of atherosclerosis. Studies in different populations, including a meta-analysis, suggested that high levels of circulating NAMPT are positively associated with cardiovascular disease and atherosclerosis-related metabolic phenotypes, as well as with the 10-year CVD Framingham risk score [3,6,11,21-23]. The pathophysiological basis for the association between NAMPT levels and CAD is still unclear, as both cardioprotection [24-27] and deleterious effects of NAMPT on the cardiovascular system have been reported [28-33].

In the present investigation, we confirmed the association of high plasma NAMPT levels with CAD in the subset of subjects with type 2 diabetes of the Brazilian cohort, while no association with CAD was observed in non-diabetic subjects. We have no clear explanation for these heterogeneous results. The mechanisms of possible interactions between NAMPT levels and glycemic status on the risk of CAD cannot be assessed in our study, given its observational nature. However, it is noteworthy that non-diabetic subjects in the highest tertile of plasma NAMPT levels had a more favorable profile of other cardiovascular risk factors, compared to subjects in the lowest tertile. They had lower BMI, lower levels of HbA1c, LDL cholesterol and triglycerides, and higher levels of total adiponectin. Adiponectin was shown to have protective effects in the initiation and progression of atherosclerosis by means of direct anti-inflammatory and anti-atherogenic mechanisms [34-36]. So it is possible to speculate that higher adiponectin levels observed in non-diabetic subjects would protect against the deleterious effects of higher NAMPT levels. No differences in other cardiovascular risk factors by NAMPT tertiles were observed in diabetic subjects, except for levels of triglycerides. Interestingly, de Luis and coworkers also observed an inverse correlation of NAMPT levels with weight, fat mass and level of triglycerides in Spanish non-diabetic obese subjects [2]. However, in that study higher NAMPT levels were associated with higher LDL-cholesterol and lower adiponectin levels. Differences in ethnicity, glycemic and/or ponderal status and use of medications in different studies could explain these heterogeneous results.

We observed an association of the T-allele of rs9770242 SNP with CAD in the Brazilian cohort. There was trend towards higher plasma NAMPT levels in carriers of the risk T-allele, but the allelic association with CAD was independent of plasma NAMPT levels. It is noteworthy that the same allele was shown to be associated with higher insulin and glucose plasma levels and increased visceral/subcutaneous fat *NAMPT* mRNA expression ratio [14,15]. NAMPT is located both intracellularly and extracellularly [37]. The intracellular pool of NAMPT may better reflect NAMPT effects on tissues than the extracellular pool [38]. Moreover, it is unclear whether intracellular NAMPT reflects plasma NAMPT concentrations, or if does not, whether there is a genotype-related effect on NAMPT intracellular concentrations. Descriptions of genotype-related effect on plasma NAMPT levels are still scarce. For instance, Wang and co-authors showed that the C allele of the -1535C > T polymorphism (rs61330082) in the *NAMPT* gene was associated with proinflammatory status and increased NAMPT levels [39].

The allelic association was not replicated in the North-American cohort. Differences in cohort composition could account for these heterogeneous genetic results. Subjects in the Brazilian cohort, individually and/or as a group, had mixed ethnic background, while the North-American cohort was composed of non-Hispanic white subjects only. Moreover, the Brazilian cohort included both subjects with or without type 2 diabetes, whereas North-American cohort included only subjects with type 2 diabetes. Selection criteria of subjects without CAD were also different in both cohorts. It was based on coronary angiography in the Brazilian cohort, whereas it was based on clinical data in the North American cohort. There was a slightly excess of heterozygotes and a deficit of GG homozygotes among CAD patients in the North-American cohort, resulting in a nominally significant deviation from Hardy-Weinberg equilibrium (HWE) in this group (Table 3 footnote). As we carefully excluded the possibility of obvious genotyping errors, the lack of HWE could be due to chance or may be a sign of depletion of the protective GG genotype from cases, which would be consistent with the association of the allele T with CAD in the Brazilian sample. Thus, we cannot completely exclude the possibility that the absence of allelic association with CAD in the North-American cohort may reflect a type 2 error (false negative results). Finally, we cannot also exclude the possibility that allelic association with CAD in the Brazilian cohort may only reflect a type 1 error (false positive results) due to population stratification. Data from the literature on *NAMPT* genotypes and CAD-related traits are scarce. A study in a Swedish cohort reported an association with myocardial infarction of the G allele of the rs1319501, a promoter SNP that is in complete linkage disequilibrium with rs9770242 [40]. Another study, but in a very particular population of Spanish patients with rheumatoid arthritis reported no association of rs9770242 with cardiovascular events or with carotid artery intima-media thickness [41]. The T allele of the rs9770242 and other SNPs in the *NAMPT* gene were shown to be associated with fasting plasma insulin and glucose levels in French-Canadian [14]. The genetic mechanism behind these allelic associations is unclear. It is noteworthy that rs9770242 is in complete linkage disequilibrium with rs1319501 in the promoter of *NAMPT*, and that the region surrounding and including rs1319501 matches the binding site for CREB (cAMP response element-binding protein) transcription factors (http://asp.ii.uib.no:8090/cgi-bin/CONSITE/consite/). The rs9770242 and other SNPs in the NAMPT gene were shown to be associated with visceral/subcutaneous *NAMPT* mRNA expression ratio in German subjects [15]. However, there was strong linkage disequilibrium between the SNPs and the functional variant was not identified.

## Conclusion

Our results suggest that high plasma NAMPT levels are associated with CAD in subjects with type 2 diabetes and that the *NAMPT* rs9770242 polymorphism may be associated with CAD in some populations. Additional studies in larger populations and based on extended haplotypes are needed to confirm the association of *NAMPT* variants with CAD.

## Abbreviations

NAMPT: Nicotinamide phosphoribosyltransferase; CVD: Cardiovascular disease; CAD: Coronary artery disease; HWE: Hardy-Weinberg equilibrium.

## Competing interests

The authors declare that they have no competing interests.

## Authors’ contributions

PSR participated in the study’s design, acquisition of data, literature review and drafted the manuscript. CSVO participated in the study’s design and acquisition of data and literature review. FC carried out the immunoassays and molecular genetic studies. FMAG helped in literature review and critically revised the manuscript for important intellectual content. VCL participated in the study’s design. JGHV helped in the analysis and interpretation of data. AD participated in the acquisition and analysis of data from the North-American cohort and critically revised the manuscript for important intellectual content. GV participated in the design of the study, performed the statistical analysis of data from the Brazilian cohort and critically revised the manuscript for important intellectual content. AFR conceived the study, participated in its design and coordination and revised the manuscript critically for important intellectual content. All authors read and approved the final manuscript.
